# The Dermatologist on Social Media: When the Pros Outweigh the Cons. Comment on “Risks and Benefits of Using Social Media in Dermatology: Cross-sectional Questionnaire Study”

**DOI:** 10.2196/31943

**Published:** 2022-02-25

**Authors:** Anthony Concilla, Melissa R Laughter, Colby L Presley, Jaclyn Anderson, Chandler W Rundle

**Affiliations:** 1 College of Osteopathic Medicine Philadelphia College of Osteopathic Medicine Philadelphia, PA United States; 2 Transitional Year Residency Dell Medical School The University of Texas at Austin Austin, TX United States; 3 Division of Dermatology Lehigh Valley Health Network Allentown, PA United States; 4 Department of Pathology School of Medicine Stanford University Stanford, CA United States; 5 Department of Dermatology Duke University Durham, NC United States

**Keywords:** Instagram, Twitter, TikTok, Facebook, internet, social media, dermatologist, generational differences, information quality, patient education, online content, risk, benefit, dermatology, cross-sectional, survey, online health information

We applaud Bressler et al [1] for their cross-sectional study determining the risks and benefits of social media use by practicing dermatologists and dermatology residents. This study found that 93.8% of survey respondents used a variety of social media sites [1]. Respondents were stratified by employment, and usage patterns and perspectives were recorded. Here, we aim to reframe the findings of Bressler et al [1] as an opportunity to encourage dermatologists to use social media to combat misinformation, serve as public health advocates, and support patients’ wellness.

While this study successfully characterizes opportunities for dermatologists to interact on social media (eg, patient education, care opportunities, improved quality of information), the gravity of these findings was not explored, as dermatologists are a significant minority of contributors to social media information. For example, Wells et al [2] found that board-certified dermatologists were responsible for only 12% (26/219) of analyzed Instagram content related to skin of color. Similarly, an analysis of psoriasis-related content on Twitter found that only 3% (17/574) of accounts belonged to dermatologists [3]. These findings show that dermatologists’ contributions pale in comparison to nondermatologists, and highlight the need for dermatologists to expand their presence on social media.

An additional, unique aspect of Bressler et al’s [1] study is the measure of dermatologists’ perspectives. The study emphasizes that dermatologists were more pessimistic than optimistic on social media use, citing perceived risks of misinformation, poor substitution of care, and increased visibility of non–evidence-based products (*P*<.001) [1]. The juxtaposition between dermatologists’ optimism and pessimism, in conjunction with a relative paucity of participation by dermatologists, is concerning. Dermatologists could embrace the opportunity to directly combat the spread of misinformation and poor patient care while simultaneously increasing access to health care, education, and up-to-date public health initiatives. Instagram, the “most valuable platform” (as determined by a single survey question), presents opportunities for interaction with the public via photos, videos, and reels. Presley et al [4], for example, recorded the metrics for the top TikTok (another video-based platform) posts and found that educational posts had the highest mean user engagement, supporting the utilization of social media for the dissemination of medical education.

Bressler et al [1] also highlight concern for professional education, privacy breaches, and the necessity of better guidelines for physicians to interact on social media. However, the American Medical Association provides guidelines, outlining that physician interactions on social media should parallel the interactions expected of them in person. Maintaining professionalism, patient confidentiality, and combating misinformation in a clear and respectful manner are pearls for physician conduct on social media platforms [5]. Users should avoid sharing or improperly storing patient health information (ie, tattoos, scars), state their conflicts of interest or affiliations, and include disclaimers with recommendations.

While dermatologists are minor contributors in the scheme of social media, it is more important than ever for this group to advocate for their patients and profession. While there are potential negatives with social media use, it is important that we recognize and face these barriers as a means to provide clear, accurate information to our patients while simultaneously providing greater access to high-quality care.
